# Tyrosine Phosphorylation of the UDP-Glucose Dehydrogenase of *Escherichia coli* Is at the Crossroads of Colanic Acid Synthesis and Polymyxin Resistance

**DOI:** 10.1371/journal.pone.0003053

**Published:** 2008-08-25

**Authors:** Soline Lacour, Emmanuelle Bechet, Alain J. Cozzone, Ivan Mijakovic, Christophe Grangeasse

**Affiliations:** 1 Institut de Biologie et Chimie des Protéines, University of Lyon, CNRS, Lyon, France; 2 Center for Microbial Biotechnology, BioCentrum, Technical University of Denmark, Lyngby, Denmark; Tufts University, United States of America

## Abstract

**Background:**

In recent years, an idiosyncratic new class of bacterial enzymes, named BY-kinases, has been shown to catalyze protein-tyrosine phosphorylation. These enzymes share no structural and functional similarities with their eukaryotic counterparts and, to date, only few substrates of BY-kinases have been characterized. BY-kinases have been shown to participate in various physiological processes. Nevertheless, we are at a very early stage of defining their importance in the bacterial cell. In *Escherichia coli*, two BY-kinases, Wzc and Etk, have been characterized biochemically. Wzc has been shown to phosphorylate the UDP-glucose dehydrogenase Ugd *in vitro*. Not only is Ugd involved in the biosynthesis of extracellular polysaccharides, but also in the production of UDP-4-amino-4-deoxy-L-arabinose, a compound that renders *E. coli* resistant to cationic antimicrobial peptides.

**Methodology/Principal Findings:**

Here, we studied the role of Ugd phosphorylation. We first confirmed *in vivo* the phosphorylation of Ugd by Wzc and we demonstrated that Ugd is also phosphorylated by Etk, the other BY-kinase identified in *E. coli*. Tyrosine 71 (Tyr71) was characterized as the Ugd site phosphorylated by both Wzc and Etk. The regulatory role of Tyr71 phosphorylation on Ugd activity was then assessed and Tyr71 mutation was found to prevent Ugd activation by phosphorylation. Further, Ugd phosphorylation by Wzc or Etk was shown to serve distinct physiological purposes. Phosphorylation of Ugd by Wzc was found to participate in the regulation of the amount of the exopolysaccharide colanic acid, whereas Etk-mediated Ugd phosphorylation appeared to participate in the resistance of *E. coli* to the antibiotic polymyxin.

**Conclusions/Significance:**

Ugd phosphorylation seems to be at the junction between two distinct biosynthetic pathways, illustrating the regulatory potential of tyrosine phosphorylation in bacterial physiology.

## Introduction

In bacteria, protein phosphorylation is catalyzed mainly by histidine-kinases which are key enzymes of the so-called “two component systems” [1], by proteins of the phosphotransferase system involved in sugar transport and phosphorylation as well as many regulatory functions and by Hanks-type Serine/Threonine Protein Kinases (STPKs) [2]. However, the presence of tyrosine-kinases has been proven in several bacterial species, and suggested in many more by homology-based gene annotation. These tyrosine-kinases share little structural similarities with their eukaryotic counterparts [3], [4] and most of them have been recently unified in a new enzyme family called BY-kinase [5]. Until now, BY-kinases have been found only in bacteria and they seem to constitute an idiosyncratic class of enzymes. They have been shown to be involved in several physiological processes such as DNA metabolism or heat shock response [6], [7]. In several bacteria, including both proteobacteria and firmicutes, they have also been established as co-polymerases involved in synthesis and export of extracellular polysaccharides [8], [9]. However, their accurate functions remain poorly understood due to slow progress in structural characterization and to the fact that only few phosphorylation substrates have been detected. BY-kinases are autophosphorylating enzymes and they are also able to phosphorylate endogenous proteins. It was only recently that sugar-dehydrogenases or -transferases involved in polysaccharide production [10]–[13], RNA polymerase sigma factors [7] and single-stranded DNA binding proteins [14] were identified as BY-kinase substrates. Nevertheless, recent phosphoproteomic studies indicate that BY-kinases could phosphorylate a significant number of other proteins [15], [16].

*Escherichia coli* produces two BY-kinases, Wzc and Etk. They are respectively encoded by genes located at approximately 46 min and 22 min on the *E. coli* chromosome in two gene-clusters both involved in the biosynthesis of extracellular polysaccharides [17], [18]. Accordingly, Wzc and Etk have been characterized as polysaccharide co-polymerases (PCP) belonging to multiprotein transmembrane machineries involved in synthesis and/or export of extracellular polysaccharides, and their autophosphorylation on several tyrosines has turned out to be a key feature in the production of these compounds [19]. In addition, it has been demonstrated that Wzc and Etk are involved in other processes. For instance, Wzc is able to phosphorylate and down regulate the activity of Int, the integrase of coliphage HK022 [20]. In the same way, Etk has been found to be involved in heat shock response by phosphorylating sigma and anti-sigma factors [7].

Protein Ugd of *E. coli*, a UDP-glucose dehydrogenase, is one of the first identified substrates of a BY-kinase [10]. Indeed, it has been shown that Ugd is phosphorylated *in vitro* by the BY-kinase Wzc. Other types of sugar-dehydrogenases, including the UDP-glucose dehydrogenase of *Bacillus subtilis* Ugd_Bs_ [21] and the UDP-acetyl-mannosamine dehydrogenase CapO of *Staphylococcus aureus* [12], have recently been shown to be tyrosine-phosphorylated. Thus, it could be speculated that tyrosine phosphorylation of this class of enzymes is a common regulatory mechanism found in several bacteria. Ugd produces UDP-glucuronic acid (UDPGA) that is a precursor and an essential component in the biosynthesis of bacterial polysaccharides, notably of *E. coli* [19]. UDPGA is found in several capsular polysaccharides (K-antigens) and in colanic acid (M-antigen), an extracellular polysaccharide produced by many strains of *E. coli*. In addition, UDPGA participates in the production of a sugar derivative, UDP-4-amino-4-deoxy-L-arabinose (L-Ara4N) which is a crucial element in bacterial resistance to antibiotics such as polymyxin and cationic peptides of the innate immune system [22]–[25]. Thus, in the search of the role of tyrosine phosphorylation in bacteria, the question was raised whether Ugd phosphorylation could affect, on the one hand, the production of polysaccharides and, on the other hand, bacterial resistance to cationic peptides. In *E. coli* K-12, we had previously demonstrated that Wzc autophosphorylation influences the production of colanic acid, [26]. In addition, we had also shown that an *etk* knock-out mutant is much less resistant to polymyxin than a wild-type *E. coli* K-12 strain. [4], [27]. However, Ugd has not been described as being phosphorylated by Etk. Therefore, it seemed particularly interesting to investigate if Ugd is also a phosphorylation substrate for Etk and if Ugd phosphorylation by either Wzc or Etk affects colanic acid production or polymyxin resistance, respectively.

In this work, we provide *in vivo* evidence that, depending on the growth conditions, Ugd can be phosphorylated by either Wzc or Etk. We show that Ugd is phosphorylated on the same site by both BY-kinases, Wzc and Etk. We further show that Wzc-mediated phosphorylation of Ugd specifically affects the biosynthesis of colanic acid whereas resistance to the cationic antimicrobial peptide polymyxin is dependent upon Ugd phosphorylation by Etk. These data represent the first report of a bacterial protein phosphorylated by two distinct tyrosine-kinases. They contribute to define the role of tyrosine phosphorylation in bacteria and provide a basis for an emerging regulatory network in *E.coli*.

## Results

### Ugd is phosphorylated *in vivo* both by Wzc and Etk

**Figure 1:**
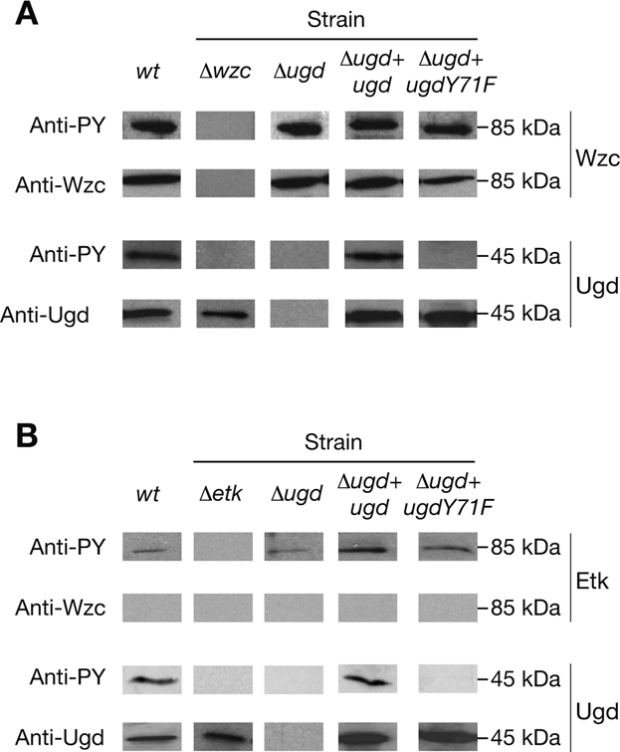
Expression and *in vivo* phosphorylation of Ugd, UgdY71F and Wzc or Etk. Western immunoblot of whole-cell lysates prepared when *E. coli* is grown under conditions allowing (A) colanic acid production or (B) polymyxin resistance were probed with either a Wzc-specific monoclonal antibody (Anti-Wzc), or a Ugd-specific monoclonal antibody (Anti-Ugd), or an anti-phosphotyrosine antibody (Anti-PY). *E. coli* wild-type strain (*wt*), *wzc*- or *etk*-deficient strain (Δ*wzc* or Δ*etk*), *ugd*-deficient strain (Δ*ugd*) and *ugd*-deficient strain complemented with wild-type *ugd* (Δ*ugd*+*ugd*) or mutated *ugd* (Δ*ugd*+*ugdY71F*).

We have previously reported that Ugd is monophosphorylated *in vitro* by the catalytic domain of Wzc, Wzc_cyto_ [10] (Table S1). The Wzc protein is not synthesized in standard growth conditions and to characterize *in vivo* phosphorylation of Ugd by Wzc, we used a strain that encodes the transcriptional regulator RcsA [26]. RcsA is known to enhance the expression of *wzc* and *ugd* [28], [29] (Table S1) but not that of *etk*, which is not expressed in our culture conditions [30], [31]. After growth in Luria Bertani medium, a total protein extract was prepared, analyzed by SDS-PAGE and immunoblotted against either a monoclonal anti-Wzc antibody, or a monoclonal anti-Ugd antibody, or an anti-phosphotyrosine antibody (Fig. 1A). Immunorevelation with the Wzc- and Ugd-specific antibodies showed signals at around 81-kDa and 45-kDa, which are respectively consistent with the expression of the two proteins. The anti-phosphotyrosine antibody revealed an expected signal corresponding to autophosphorylated Wzc. Also, we observed a 45-kDa signal that could correspond to the phosphorylated form of Ugd. To strengthen these observations, we similarly analyzed a *wzc* knock-out mutant [26]. We did not detect any signal for Wzc or phosphotyrosine around 45-kDa, whereas Ugd was still detected by the Ugd-specific antibody (Fig. 1A). In addition, the *ugd* gene was inactivated (Table S1) and the Δ*ugd* strain obtained was analyzed as described above (Fig. 1A). We still observed the expression and phosphorylation of Wzc, but no 45-kDa signal was detected by either the Ugd-specific antibody or the anti-phosphotyrosine antibody. These data confirmed that Ugd was phosphorylated *in vivo* on tyrosine by Wzc.

*E. coli* cells encode another BY-kinase, namely Etk, that is homologous to Wzc. We wondered whether Etk was also able to phosphorylate Ugd. Like Wzc, Etk is not produced by *E. coli* K-12 under standard laboratory growth conditions. In addition, *etk* expression is not dependent on protein RcsA. It has previously been shown that *etk*, but not *wzc*, is expressed when *E. coli* K-12 grows in a culture medium at low pH and low concentration of magnesium and iron ions. Such conditions induce resistance to cationic antimicrobial peptides [27]. We verified that *etk* was expressed during growth of *E. coli* K-12 in such medium. No 81-kDa signal was observed when detection was carried out with the Wzc-specific antibody whereas a signal appeared at this position when we used the anti-phosphotyrosine antibody (Fig. 1B). This observation confirmed that *wzc* is not expressed and strongly suggested that autophosphorylated Etk is produced under these culture conditions. To validate this point, we analyzed an *etk*-deficient strain. Immunoblot analysis showed no 81-kDa signal and supported the assumption that the signal detected with the anti-phosphotyrosine antibody in the wild-type strain corresponded to Etk autophosphorylation. In addition, RT-PCR experiments were performed and we observed that the *etk* gene was effectively expressed under these growth conditions (data not shown). To assess if Etk can phosphorylate Ugd, we also analyzed total protein extracts of wild-type, *ugd*-deficient and *etk*-deficient strains of *E. coli* by immunorevelation with the Ugd-specific antibody and the anti-phosphotyrosine antibody (Fig. 1B). We observed that Ugd was produced and phosphorylated in the wild-type strain. On the contrary, no 45-kDa phosphorylation signal was detected with *ugd* and *etk* knock-out mutants while Ugd was still detected in the *etk* mutant. These observations confirmed that Ugd was also phosphorylated *in vivo* by Etk.

### Ugd is activated by phosphorylation on Tyr71

To decipher the role of Ugd phosphorylation, it was necessary to identify the site of phosphorylation of *E. coli* Ugd. We had already observed that Ugd seemed to be monophosphorylated [10]. Mass spectrometry analysis (MALDI-TOF) failed to identify the Ugd phosphorylated tyrosine probably because of the low occupancy of bacterial phosphorylation sites [32]. Therefore, sequence alignments were performed with protein Ugd_Bs_, a UDP-sugar-dehydrogenase from *Bacillus subtilis* homologous to Ugd and also phosphorylated on tyrosine (Fig. S1A). Several tyrosines of Ugd (Tyr10, Tyr150, Tyr249, Tyr335 and Tyr380) that seemed conserved in Ugd_Bs_ were mutated to phenylalanine. However, when each mutant protein was purified and phosphorylated *in vitro* by the catalytic domain of Wzc, Wzc_cyto_, a radioactive signal corresponding to phosphorylated Ugd was still detected for each of them (Fig. S1B). While this work was in progress, a study of the *B. subtilis* phosphoproteome indicated that Ugd_Bs_ was phosphorylated *in vivo* on tyrosine 70 [16]. According to sequence alignments, the closest tyrosine in the *E. coli* Ugd sequence is Tyrosine 71 (Tyr71) (Fig. S1A). We hypothesized that Tyr71 could be the phosphorylation site of Ugd and we constructed the Ugd mutant Tyr71Phe (UgdY71F).

**Figure 2:**
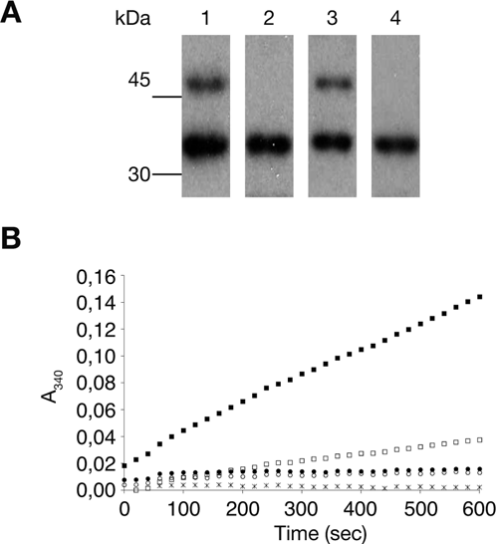
Activation of the UDP-glucose dehydrogenase activity of Ugd by phosphorylation on Tyr71. (A) Autoradiography of SDS-PAGE on which reaction mixtures containing [γ-^32^P]ATP and either Ugd and Wzc_cyto_ (lane 1) or UgdY71F and Wzc_cyto_ (lane 2) or Ugd and Etk_cyto_ (lane 3) or UgdY71F and Etk_cyto_ (lane 4) were analyzed. (B) UDP-glucose dehydrogenase activity was monitored at 340 nm for 10 min by measuring NADH formation: Ugd (□), Ugd previously phosphorylated by Wzc_cyto_ (▪), UgdY71F (○) and UgdY71F previously incubated with Wzc_cyto_ (•). As a control, a reaction mixture without Ugd was used (*). Standard deviations are not indicated because of low variations.

To check this hypothesis, the phosphorylation signal of Ugd was compared with that of UgdY71F. When incubated for 2 min with Wzc_cyto_ (kinase/substrate ratio of 1/100), we observed that Ugd was phosphorylated whereas no radioactive signal was detected for UgdY71F (Fig. 2A). These data suggested that Tyr71 is the phosphorylation site of Ugd. To check that the folding of the Ugd protein was not affected by the Y71F mutation, dynamic light scattering (DLS) measurements were performed. The hydrodynamic radius measured for Ugd and UgdY71F were the same (*R_S_* = 62+/−3 Å) confirming that the overall structure of Ugd was most likely not affected by the Tyr to Phe mutation. To confirm Tyr71 as the phosphorylation site responsible for activating the enzyme, the dehydrogenase activity of Ugd and UgdY71F was measured with or without prior incubation with Wzc_cyto_ and ATP (Fig. 2B). We have previously shown that Ugd phosphorylation increased its dehydrogenase activity [10]. As expected, Ugd activity was stimulated more than 10-fold upon phosphorylation. By contrast, UgdY71F activity remained unaffected by incubation with Wzc_cyto_. In addition, UgdY71F was less active than Ugd which is in agreement with our previous observations showing that Ugd purified from *E. coli* is partially phosphorylated, and that its extensive dephosphorylation by the phosphotyrosine phosphatase Wzb reduces its activity [10]. These data indicated that UgdY71F was no longer activated by phosphorylation and we concluded that Tyr71 was the regulatory phosphorylation site of Ugd.

### Ugd phosphorylation by Wzc on Tyr71 influences the production of colanic acid

The *cps* operon that includes *wzc*, and the *ugd* gene that is located elsewhere on the genome, are required for the synthesis of colanic acid, the extracellular polysaccharide of *E. coli* K-12. Expression of both *cps* operon and *ugd* are dependent on the Rcs system [29] and, as previously shown, the strain producing the transcriptional regulator RcsA is able to synthesize colanic acid [26]. In this strain, we showed that Ugd is phosphorylated *in vivo* by Wzc (Fig. 1A). We therefore used this strain (referred to herein as the wild-type) to assess the importance of Ugd phosphorylation on colanic acid synthesis. First, we confirmed that Ugd Tyr71 was not phosphorylated *in vivo* by Wzc. For this, we complemented the *ugd*-deficient strain (Δ*ugd* strain) with an episomal copy of either the *ugd* gene (Δ*ugd*+*ugd* strain) or the *ugd* Y71F allele (Δ*ugd*+*ugdY71F* strain) (Table S1). Total protein extracts were prepared from these two strains and the protein synthesis and phosphorylation for both Wzc and Ugd were analyzed by antibodies as described above (Fig. 1A). We observed that proteins Wzc, Ugd, and UgdY71F, were produced. In contrast, Ugd was phosphorylated only in the strain Δ*ugd*+*ugd*. Indeed, no 45-kDa phosphorylation signal was detected for the strain producing UgdY71F. These data confirmed that *in vivo* phosphorylation of Ugd by Wzc occurs at Tyr71.

**Figure 3:**
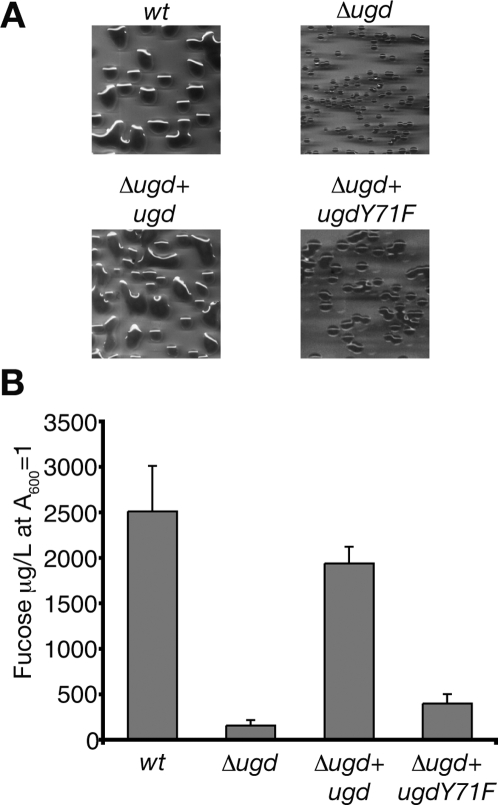
Influence of Ugd Tyr71 phosphorylation on colanic acid production. (A) Colony morphology of *E. coli wt* and mutants. Photographs were taken after growth on LB Agar plates for 24 h at 37°C. (B) Production of colanic acid by wild-type *E. coli* and mutants. The amount of colanic acid was determined in each strain by measuring fucose, and was expressed as µg/L of culture of A_600_ = 1. Standard deviations from four independent experiments are indicated with error bars.

*E. coli* K-12 producing colanic acid exhibits a mucoid phenotype, and on plates, colonies have a fatty and shiny appearance. In contrast, smaller and duller colonies are observed when colanic acid is not produced. Therefore, we compared the colony morphology of strains producing Ugd, wild-type form or mutated on Tyr71 (Fig. 3A). We first observed that colony morphologies of the wild-type and Δ*ugd*+*ugd* strains were almost indistinguishable and they had characteristics of a mucoid phenotype due to the production of colanic acid (Fig. 3A). By contrast, colonies formed by the Δ*ugd* strain differed considerably and were not mucoid. When looking at the colony morphology of Δ*ugd*+*ugdY71F* strain, we also observed a non-mucoid phenotype similar to the Δ*ugd* strain (Fig. 3A). These data supported the hypothesis that the Wzc-mediated phosphorylation of Ugd on Tyr71 controls colanic acid production. To strengthen this observation, we prepared and quantified colanic acid produced by each strain used (Fig. 3B). Quantification was performed by measuring the amount of fucose, which is exclusively found in this polysaccharide [17]. As expected, the wild-type and Δ*ugd*+*ugd* strains produced comparable amounts of colanic acid, whereas the amount of colanic acid determined for the Δ*ugd* strain was up to 10-fold lower. Concerning the Δ*ugd*+*ugdY71F* strain, a 5-fold reduction was measured compared to Δ*ugd*+*ugd* strain. Colanic acid still produced by the Δ*ugd*+*ugdY71F* strain was likely due to the basal activity of UgdY71F (Fig. 2B). Accordingly, colony morphology of Δ*ugd*+*ugdY71F* strain began to be mucoid when growth was allowed for more than 48 hours (data not shown). These data nevertheless confirm the two distinct phenotypes: mucoid for the wild-type and Δ*ugd*+*ugd* strains, and non-mucoid for the Δ*ugd* and Δ*ugd*+*ugdY71F* strains. They also confirm that phosphorylation of UgdTyr71 by Wzc affects colanic acid production.

### Ugd phosphorylation by Etk on Tyr71 influences polymyxin resistance

In *E. coli* K-12, Ugd catalyzes the first step of the biosynthesis of L-Ara4N, which confers bacterial resistance to cationic antimicrobial peptides and antibiotics such as polymyxin [33]. It has been previously reported that *E. coli* resistance to polymyxin depends also on the expression of *etk* [4], [27]. Here, we showed that Ugd was phosphorylated *in vivo* by Etk when *E. coli* was grown under culture conditions allowing resistance to polymyxin [34] (Fig. 1B). Therefore, it was tempting to assume that Etk could regulate polymyxin resistance by phosphorylating Ugd. We therefore tested whether Etk-catalyzed *in vivo* phosphorylation of Ugd would also occur on Tyr71. For this, the Δ*ugd*+*ugd* and the Δ*ugd*+*ugdY71F* strains were grown in the conditions allowing polymyxin resistance and total protein extracts were prepared and analyzed by SDS-PAGE and immunoblotting. As shown in Fig. 1B, detection with the Ugd-specific antibody indicated that Ugd was produced in both Δ*ugd*+*ugd* and Δ*ugd*+*ugdY71F* strains. However, when immunoblotted with the anti-phosphotyrosine antibody, a phosphorylation signal was detected only when the wild-type form of Ugd was produced. This observation confirmed that Ugd was specifically phosphorylated *in vivo* on Tyr71 by Etk and that Ugd Tyr71 phosphorylation could consequently influence polymyxin resistance.

**Figure 4:**
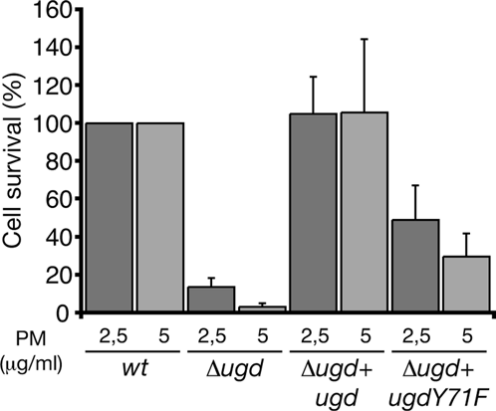
Influence of Ugd Tyr71 phosphorylation on polymyxin resistance. Polymyxin resistance of *E. coli* mutants are expressed relatively to the survival of wild type taken as 100%. Resistance was measured in the presence of a polymyxin concentration of either 2.5 or 5 µg/ml. Standard deviations from six independent experiments are indicated with error bars.

To check this, each strain was assayed for polymyxin resistance by measuring the percentage of surviving cells (Fig. 4). *E. coli* strains were grown in the culture medium allowing resistance to polymyxin, incubated for 1 hour in the presence of varying concentrations of polymyxin and plated onto agar-LB plates. Survival rates are expressed as the percentage of the number of colonies formed with a strain grown in the presence of polymyxin with respect to the number of colonies obtained with the same strain grown in the absence of the antibiotic. Optimal resistance of the wild-type strain was 39% or 25% survival in the presence of 2.5 or 5 µg/ml polymyxin, respectively. As expected, the Δ*ugd* strain exhibited 7% to 1% survival, which represents only 17% to 3% of the wild-type strain resistance (Fig. 4). The Δ*ugd*+*ugd* strain survival values were not significantly different from those of the wild-type strain. By contrast, the strain expressing UgdY71F showed a survival level decreased over 2 and 3-fold depending on the polymyxin concentration (Fig. 4). We therefore conclude that Ugd phosphorylation by Etk on Tyr71 participates in polymyxin resistance.

## Discussion

**Figure 5:**
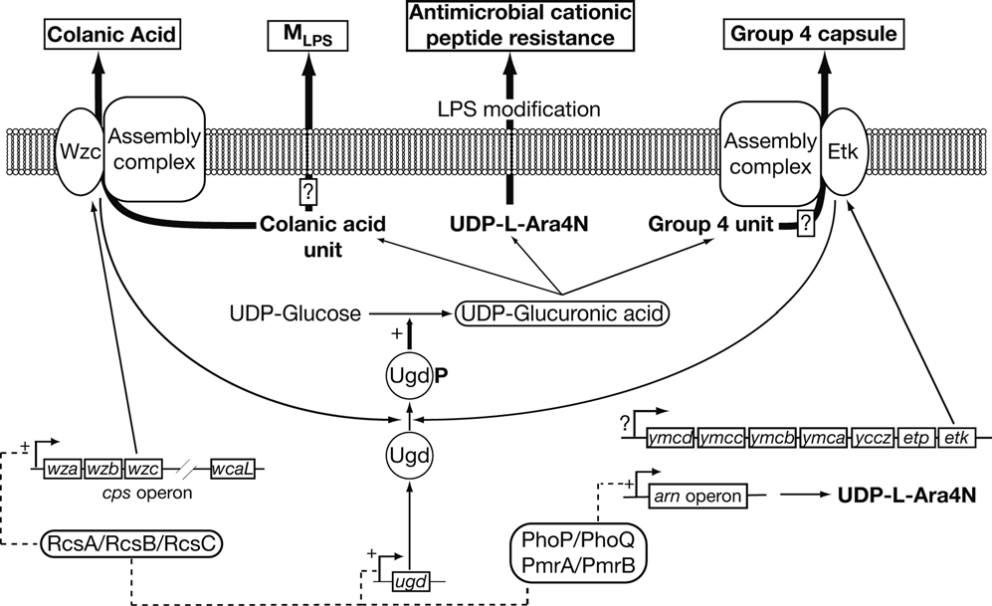
Schematic model for Ugd phosphorylation by Wzc or Etk as control element for extracellular polysaccharides production and resistance to antimicrobial cationic peptide. The two-component systems PhoP/PhoQ and PmrA/PmrB, or the RcsA/RcsB/RcsC system alone allow expression of genes involved in L-Ara4N production (*arn* and *ugd*) or colanic acid biosynthesis (*cps* operon and *ugd*), respectively. In both situations, phosphorylation of Ugd by Wzc and/or Etk influences UDP-glucuronic acid production and consequently the two phenotypes. Since, Ugd is involved in M_LPS_ and Group 4 capsule synthesis, it is assumed that Ugd phosphorylation could also influence their production (boxed question mark symbols). A question mark symbol is also used to indicate that the mechanisms governing the expression of the *ymc* operon are still unknown.

In this work, we showed that Ugd of *E. coli* is phosphorylated on tyrosine 71 by the BY-kinases Wzc and Etk. This finding constitutes the first example of a bacterial protein phosphorylated on tyrosine by two distinct BY-kinases (Fig. 5). In *E. coli*, so far only 3 proteins have been characterized as being phosphorylated by Etk or Wzc [7], [20]. However, no less than 7 other proteins have recently been found to be tyrosine phosphorylated in *E. coli* cells grown under standard laboratory conditions [15]. These proteins carry out functions as diverse as tRNA synthesis, transport of amino acids, protein translation and stress response. Since, Wzc and Etk are the only tyrosine kinases characterized in *E. coli*, it is possible that they phosphorylate these proteins. Alternatively, presently unidentified tyrosine kinases could be present in *E. coli*. To support the first hypothesis, it has been demonstrated that the colanic acid operon (*cps*), which includes 19 genes, contains a stem-loop transcriptional attenuator which is located immediately after the third gene, namely *wzc* [35]. *wzc* might therefore be expressed independently of the other *cps* genes and be involved in the phosphorylation of other proteins so as to regulate other cellular functions. There are also arguments for the second hypothesis, namely that other tyrosine kinases could be encoded by the genome of *E. coli*. Several proteins of unknown function harbor the Walker A and B motifs [36] that constitute the active sites of Wzc and Etk, and they might function as tyrosine kinases. Furthermore, Zheng and coworkers have recently reported that the protein of unknown function YihE indeed exhibits a eukaryotic-like kinase fold despite sharing no sequence homology with eukaryotic kinases [37]. This finding supports the existence of still unknown bacterial kinases that would harbor a new type of phosphorelay mechanism. Be that as it may, the role of BY-kinases in phosphorylating other proteins seems underestimated and new regulatory networks based on tyrosine phosphorylation are likely to exist not only in *E. coli* but also in a large number of proteobacteria and firmicutes in which BY-kinases have been identified [5].

We established that Wzc-mediated phosphorylation of Ugd influences the production of colanic acid (Fig. 5). It has already been demonstrated that the phosphorylation-dephosphorylation of BY-kinases is involved in the biosynthesis of extracellular polysaccharides in various bacteria and more precisely in the assembly or the export of the nascent polysaccharide [26], [38]–[42]. The function of BY-kinases has been mainly studied by performing mutations in their catalytic site. Therefore, one might assume that, in addition to an effect due to the phosphorylation-dephosphorylation process of a BY-kinase itself, an effect due to the altered phosphorylation of a potential substrate might also influence the biosynthesis of the polysaccharide. Ugd, that is involved in the biosynthesis of the repeat unit of colanic acid, is in line with this idea (Fig. 5) [17]. Therefore, colanic acid biosynthesis is controlled by phosphorylation at two levels: the assembly and export of the polyssacharide (Wzc phosphorylation) and the synthesis of the colanic acid repeat unit (Ugd phosphorylation). This analysis could be extended to other bacteria. For instance, Minic and co-workers have described the phosphorylation of the UDP-glycosyl-transferase EpsE, which is involved in the production of the expolysaccharide of *Streptococcus thermophilus*, by the BY-kinase EpsD [13].

In the particular case of *E. coli* K-12, the phosphorylation of Ugd could also have side effects on the production of other polysaccharidic compounds. A recent study has demonstrated that variation of polysaccharide chain length depends on the UDP-glucose (UDPG) concentration available in the bacterial cell [43]. In this work, we did not observe such a variation of the colanic acid polymer length (data not shown) even though the UDPG/UDPGA ratio might vary in accordance with the Ugd phosphorylation level. Also, UDPGA is involved in the biosynthesis of certain Group 4 capsules (G4C) produced by some pathogenic strains of *E.coli* [44]. G4C are well-established virulence factors and require Etk to be secreted [18], [30]. Accordingly, Etk-mediated phosphorylation of Ugd could influence the production of G4C (Fig. 5). Similarly, Meredith and co-workers have reported that colanic acid repeats could modify the lipopolysaccharide (LPS) of *E. coli* K-12, forming thus a novel LPS glycoform henceforth called M_LPS_ [45]. The biosynthesis of M_LPS_ could also be affected by phosphorylation of Ugd (Fig. 5). Therefore, tyrosine phosphorylation of Ugd, and more generally of enzymes involved in polysaccharide biosynthesis, could provide keys to understand the biosynthesis of polysaccharide which might be far more complex than presently believed.

We also demonstrated that Etk-mediated phosphorylation of Ugd is connected with *E. coli* resistance to polymyxin (Fig. 5). The *etk* gene is located in the *ymc* operon which is thought not to be expressed in *E. coli* K-12 strain grown in LB medium, but only in pathogenic *E. coli* strains [18]. Indeed, an insertion sequence *IS1* is found in the promoter of the *ymc* operon in *E. coli* K-12 but not in pathogenic *E. coli* strains. Therefore, our results raise the question of the regulation of *etk* expression in *E. coli* K-12. We have previously shown that an *etk*-deficient strain of *E. coli* is unable to resist to polymyxin [27]. This finding has recently been confirmed by a report indicating that the kinase activity of Etk is required *per se* for polymyxin resistance [4]. In addition, an *etk* knock-out mutant of an *E. coli* K-12 strain has been reported to be altered in its heat shock response [7]. These observations demonstrate that *etk* is expressed in *E. coli* K-12 in spite of the *IS1* sequence, at least under some particular growth conditions. No data have been reported concerning the expression of the *ymc* operon (Fig. 5) but, as *etk* is the last gene of this operon, one might suggest that a cryptic promoter could be involved in *etk* specific expression. More likely, it could be speculated that the *IS*1 insertion could have a positive transcriptional effect under particular conditions. For example, genomic transposition within the regulatory locus *bglR* constitutes the major class of activating mutations that enable transcription of the *bgl* operon, which is silent in wild-type *E. coli* strains under laboratory conditions [46].

Colanic acid synthesis or polymyxin resistance depend each on two distinct sets of proteins, that include respectively Wzc or Etk, and that are synthesized under specific conditions (Fig. 5). Beside the influence of Ugd phosphorylation, one cannot preclude that other events would affect those biological processes. To illustrate this, it has been shown that Wzc expression does not complement polymyxin resistance of an *etk*-deficient strain [27]. Similarly, Wzc proteins from the *E. coli* K12 and K30 strains are also not interchangeable because of specific interactions between each Wzc proteins and their cognate capsule assembly complex [47]. Therefore, we assume that Wzc and Etk themselves are not likely only crucial in Ugd phosphorylation, but also in establishing interactions with other proteins involved in colanic acid synthesis and polymyxin resistance. Another possibility is that Etk and Wzc would likely phosphorylate other proteins [15]. Therefore, one cannot exclude that Wzc and/or Etk would specifically phosphorylate other endogenous proteins involved in colanic acid production or polymyxin resistance. For instance, WcaJ protein is involved in colanic acid synthesis in *E. coli* K12 [17] and its homolog in *S. thermophilus*, EpsE, is phosphorylated on tyrosine by the BY-kinase EpsD [13]. Therefore, Wzc-mediated phosphorylation of WcaJ would also influence colanic acid synthesis.

One can speculate that certain environments would induce simultaneous both expression of colanic acid and polymyxin resistance. At this moment, some factors could also participate in determining whether colanic acid or polymyxin resistance is expressed. The presence of these factors could depend on activation of the Rcs, PmrA/PmrB and PhoP/PhoQ two-components sytems, that govern the expression of the *cps* and *arn* operons (Fig. 5), but that are also known to regulate other numerous cellular activities [48], [49]. For instance, it has already been brought up that BY-kinases, namely Wzc and Etk, could act as membrane receptors, capable of sensing input signals, thereby affecting their kinase activity and controlling signal transduction [5], [50]. In line with our hypothesis, some factors produced by either the Rcs, or PmrA/PmrB or PhoP/PhoQ systems could influence specifically Etk or Wzc kinase activity or their ability to function in colanic acid synthesis and polymyxin resistance.

Complicated as it may, both high-throughput phosphoproteomic studies [50] and structural characterization of Wzc and Etk [4] will be helpful to generate valuable data to understand further their biological role in *E. coli*. More generally, our data represent a step toward deciphering the regulatory role of tyrosine phosphorylation in bacteria and illustrate that bacterial protein phosphorylation networks could be more complex than initially expected.

## Methods

### Bacterial strains

Strains and plasmids used in this study are listed in Table S1. *E. coli* JM83, wild type or mutated, was used to perform experiments on colanic acid production. *E. coli* W3110, wild type or mutated, was used to perform polymyxin resistance assay. *E. coli* XL1-Blue strain was used to propagate plasmids in cloning experiments. Bacteria were grown in LB medium at 37°C or in the medium inducing polymyxin resistance (see below). Antibiotics were added at the following concentrations: 50 µg/ml ampicillin, 25 µg/ml kanamycin, 15 µg/ml tetracyclin.

### Gene disruption, mutagenesis and cloning

Gene replacement of *ugd* in *E. coli* W3110 or JM83 strain was performed by one-step inactivation as previously described [51]. Here, *ugd* was replaced with the kanamycin resistance cassette (Kan^R^) (Table S1). Two-way mutagenic PCR was performed to substitute Ugd tyrosines to phenylalanines. For *in vivo* analysis of colanic acid production and polymyxin resistance, *ugd* and *ugdY71F* were expressed after cloning either in the pUC-*rcsA* plasmid opened with *Sac*I and *Acc*65I, or in the pUC plasmid opened with *Acc*65I and *Bam*HI, respectively. The 840-bp *etk* fragment encoding the cytoplasmic domain of Etk (amino acids 447–726) was cloned into the pQE30 vector previously opened with *Bam*HI and *Hin*dIII. Constructs were checked by DNA sequencing. Primers used in this study are described in Table S2.

### RNA manipulation

Total RNA was prepared from *E.coli* cells collected in the postexponential phase of growth. RNA was purified with the High Pure RNA isolation kit (Roche). Contaminating DNA was removed by additional treatment for 20 min at 37°C with 10 U DNase I (Roche). RT-PCR amplification was carried out with the SuperScript II reverse Transcriptase (Invitrogen) following the manufacturer's recommendations. A control sample without reverse transcriptase was included to confirm the absence of contaminating DNA.

### Protein purification, kinase assay and UDP-glucose dehydrogenase assay

Wzc_cyto_, Etk_cyto_, Ugd or UgdY71F were expressed in *E. coli* XL1-Blue cells. The purification procedure and *in vitro* kinase assays were performed as previously described [10]. For kinase assays, 1 µg of protein Ugd was incubated for 30″ to 15 min at 37 °C with varying amount of Wzc_cyto_ ranging from 0.002 µg to 1 µg. The reaction mixtures were then analyzed by SDS-PAGE. After electrophoresis, gels were soaked in 20% TCA for 10 min at 90°C, stained with Coomassie blue and dried. Radioactive proteins were visualized by autoradiography using direct exposure films. Ugd dehydrogenase activity measurement were performed in a thermostated cuvette at 37 °C, on a PowerWave 340 BIO-TEK spectrophotometer as described [52].

### Dynamic light scattering (DLS) measurements

DLS measurements were performed at 18 °C in a buffer containing 50 mM NaH_2_PO_4_ pH 7.8, 300 mM NaCl, 10% glycerol and 150 mM imidazol with a Zetasizer Nano series Malvern instrument. A light path of 3 mm was used. Protein concentration was of 14 mg/mL and 8 mg/mL for Ugd and UgdY71F, respectively.

### Immunoblot analysis

Bacterial cell extracts were analyzed by Western blotting after SDS-PAGE. Proteins Wzc and Ugd were detected using specific monoclonal antibodies prepared in our laboratory according to the procedure described [53] and a goat-anti-mouse secondary antibody HRP conjugate (Biorad). Phosphorylation was detected using PY20 monoclonal anti-phosphotyrosine-HRP conjugate antibody (Sigma).

### Colanic acid purification

The method used was based on the procedure previously described [54]. 50 ml cell culture were heated for 15 min at 100 °C to denature EPS-degrading enzymes. After cooling, they were centrifuged at 13,200 ×g at 4 °C for 30 min. 40 ml of the supernatant fraction were then precipitated by addition of three volumes of ethanol. The mixture was maintained at 4°C overnight and centrifuged in the same conditions as above. The resulting pellet was dissolved in 5 ml of distilled water, dialyzed for 48 h against distilled water (Membrane MWCO 3,500 Da) and dried. Residual polypeptides were removed by precipitation with 5 ml of 10% (v/v) trichloroacetic acid and centrifuged again at 13,200 ×g at 4 °C for 30 min. The supernatant was dialyzed again for 5 days against distilled water and dried. The resulting preparation was re-suspended in 1 ml of distilled water and stored until quantification.

### Quantification of colanic acid

Quantification of colanic acid was carried out according to [55] by measuring the amount of non-dialyzable methylpentose (6-deoxy-hexose), namely fucose which is a specific component of this exopolysaccharide. 10 to 100 µl of the colanic acid preparation were diluted to 1 ml with distilled water, and mixed with 4.5 ml of H_2_SO_4_/H_2_O (6∶1 v/v). The mixture was prepared at room temperature, then heated at 100 °C for 20 min, and finally cooled down to room temperature. For each sample, absorbance at 396 nm and 427 nm was measured either directly (control sample (A_-co_)) or after addition of 100 µl of cysteine hydrochloride (cysteine sample (A_-cy_)). Indeed, biological extracts often contain compounds which under heating with H_2_SO_4_ yield brown products absorbing between 396 and 427 nm. The absorption due to this unspecific reaction was subtracted from the total absorption of the sample: A_396-co_ and A_427-co_ were respectively subtracted from A_396-cy_ and A_427-cy_ to obtain DA_396_ and DA_427_. Values of (DA_396_–DA_427_) were directly correlated to methylpentose concentration by using a standard curve obtained with a fucose concentration ranging from 5 to 100 mg/ml.

### Polymyxin resistance assay

Tested strains were grown for 12 h in LB medium at 37°C, diluted 100 fold and grown again overnight in N-minimal medium at pH 7.7, in the presence of 0.2% glucose and 10 mM Mg^2+^. Cultures were then harvested and washed three times with a N-minimal medium at pH 5.8 (mild acid conditions), in the presence of 0.2% glucose and 10 µM Mg^2+^ (low magnesium conditions) and 1 µM Fe^3+^ (Resistance-Inducing medium) [56]. Cells were diluted 1∶100 into the same medium and incubated for 4 h at 37 °C. 100,000 bacteria were thus recovered and incubated for one additional hour in the presence of a varying concentration of polymyxin B from 0.5 to 5 µg /ml at 37°C. Finally, 50 ml of the solution were plated onto LB agar plates and the number of colonies was counted after overnight aerobic incubation at 37°C. The percentage of survival (%) was calculated as follows: number of colonies of polymyxin-treated culture / number of colonies of control culture ×100.

## Supporting Information

Figure S1 Analysis of the Ugd amino acids sequence to characterize the phosphorylated tyrosine. (A) Comparison of both the amino-acid sequences and the predicted secondary structure of Ugd and YwqF. β, α, and η indicate β-sheet, α-helices and 3.10 helices, respectively. Secondary structure elements of Ugd and YwqF have been predicted using Streptococcus pyogenes UDP-glucose dehydrogenases (PDB code 1DL1) and Pseudomonas aeruginosa GDP-mannose dehydrogenase (PDB code 1MV8) as templates respectively (Gouet et al., 2003; Rost and Liu, 2003) Conserved tyrosines are indicated in cyan. Tyr70 of YwqF and Tyr71 of Ugd are highlighted in green. (B) Autoradiography of SDS-PAGE on which reaction mixtures containing [γ-32P]ATP and either Ugd and Wzccyto (lane 1), or UgdY10F and Wzccyto (lane 2), or UgdY150F and Wzccyto (lane 3), or UgdY249F and Wzccyto (lane 4), or UgdY335F and Wzccyto (lane 5), or UgdY380F and Wzccyto (lane 6) were analyzed. References 1.Gouet, P., Robert, X., and Courcelle, E. (2003) ESPript/ENDscript: Extracting and rendering sequence and 3D information from atomic structures of proteins. Nucleic Acids Res 31: 3320-3323. 2.Rost, B., and Liu, J. (2003) The PredictProtein server. Nucleic Acids Res 31: 3300-3304.
(21.88 MB TIF)

Table S1 Bacterial strains and plasmids used in this study
(0.04 MB DOC)

Table S2 Primers used in this study
(0.04 MB DOC)
