# Supplementary figures and images for: Tyrosine Phosphorylation of the UDP-Glucose Dehydrogenase of Escherichia coli Is at the Crossroads of Colanic Acid Synthesis and Polymyxin Resistance
Source: PLoS One. 2008 Aug 25;3(8):e3053. doi: 10.1371/journal.pone.0003053 (PMC2516531; doi:10.1371/journal.pone.0003053)

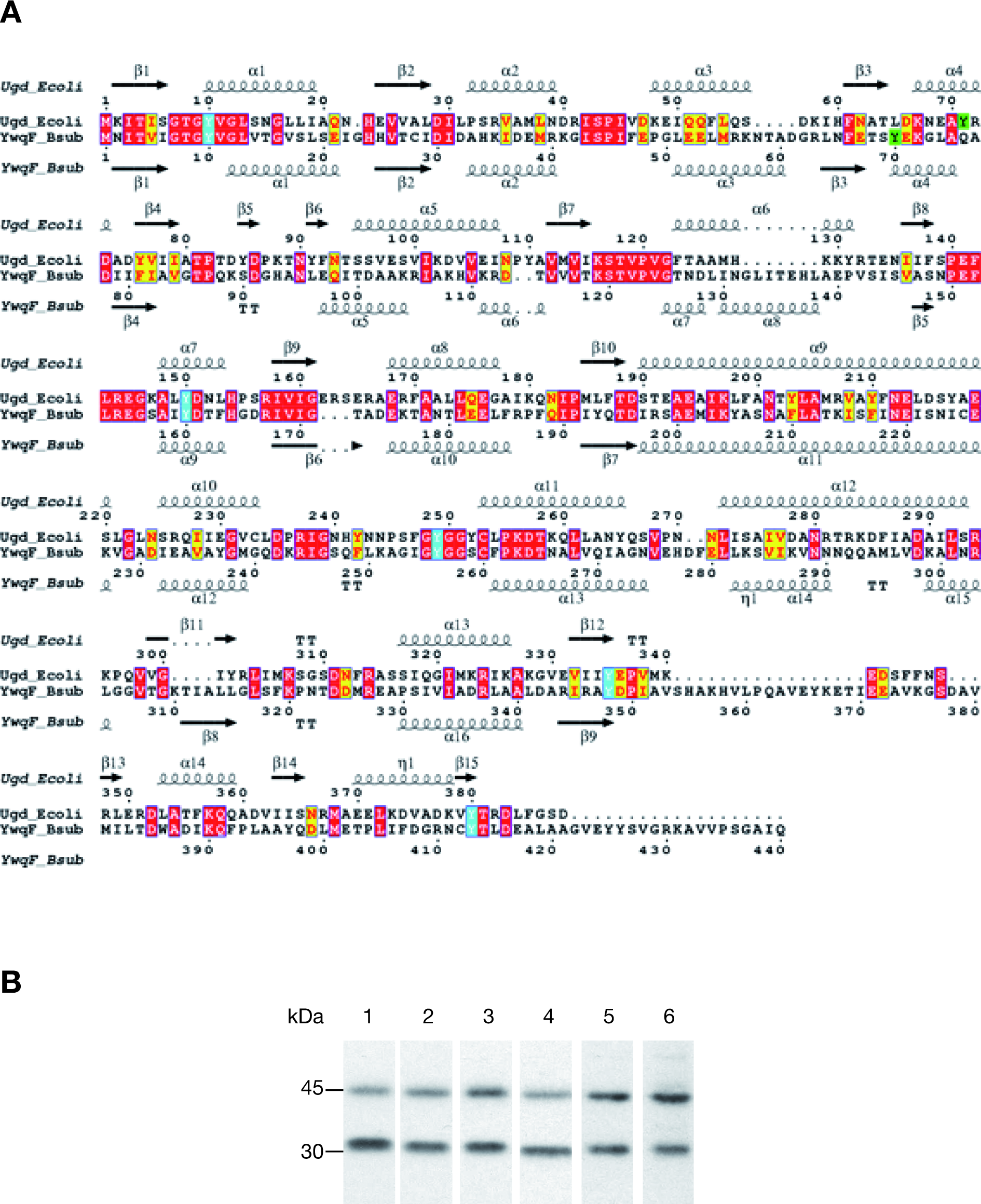

Supplement: Figure S1 — Analysis of the Ugd amino acids sequence to characterize the phosphorylated tyrosine. (A) Comparison of both the amino-acid sequences and the predicted secondary structure of Ugd and YwqF. β, α, and η indicate β-sheet, α-helices and 3.10 helices, respectively. Secondary structure elements of Ugd and YwqF have been predicted using Streptococcus pyogenes UDP-glucose dehydrogenases (PDB code 1DL1) and Pseudomonas aeruginosa GDP-mannose dehydrogenase (PDB code 1MV8) as templates respectively (Gouet et al., 2003; Rost and Liu, 2003) Conserved tyrosines are indicated in cyan. Tyr70 of YwqF and Tyr71 of Ugd are highlighted in green. (B) Autoradiography of SDS-PAGE on which reaction mixtures containing [γ-32P]ATP and either Ugd and Wzccyto (lane 1), or UgdY10F and Wzccyto (lane 2), or UgdY150F and Wzccyto (lane 3), or UgdY249F and Wzccyto (lane 4), or UgdY335F and Wzccyto (lane 5), or UgdY380F and Wzccyto (lane 6) were analyzed. References 1.Gouet, P., Robert, X., and Courcelle, E. (2003) ESPript/ENDscript: Extracting and rendering sequence and 3D information from atomic structures of proteins. Nucleic Acids Res 31: 3320-3323. 2.Rost, B., and Liu, J. (2003) The PredictProtein server. Nucleic Acids Res 31: 3300-3304. (21.88 MB TIF) [file pone.0003053.s001.tif]
