# Supplementary material for: Tyrosine Phosphorylation of the UDP-Glucose Dehydrogenase of Escherichia coli Is at the Crossroads of Colanic Acid Synthesis and Polymyxin Resistance
Source: PLoS One. 2008 Aug 25;3(8):e3053. doi: 10.1371/journal.pone.0003053 (PMC2516531; doi:10.1371/journal.pone.0003053)
Supplement: Table S1 — Bacterial strains and plasmids used in this study (0.04 MB DOC) [file pone.0003053.s002.doc]

**Table S1: Bacterial strains and plasmids used in this study**

| Strain or plasmid | **Genotype or description** | **Reference or source** |
| --- | --- | --- |
| XL1-Blue | *sup*E44 *hsdR*17 *rec*A1 *end*A1 *gyr*A46 *thi* *rel*A1 lac^-^  F’[*pro*AB^+^ *lac* I^q^ lacZΔM15 *Tn*10 (tet^R^)] | Bullock *et al*, 1987 |
| W3110 | *(rrnD-rrnG)1, rph-I, F^-^, λ^-^* | E. coli Genetic stock, Yale University |
| JM83 | F^-^ *ara* Δ(*lac-pro*AB) *rpsL* (Str^R^) [φ 80 Δ*lac*Δ(*lacZ*)M15] *thi* | Yanisch-Perron *et al*, 1985 |
| JM83 *ugd*::Km^R^ | JM83 with *ugd*::Km^R^ insertion | This study |
| W3110 *ugd*::Km^R^ | W3110 with ugd::Km^R^ insertion | This study |
| pQE30-*ugd* | Encoding Ugd from Lys^2^ to Asp^388^, cloned in *Bam*HI/*Hin*dIII sites, Amp^R^ | Grangeasse *et al*, 2003 |
| pQE30-*ugdY10F* | As pQE30-*ugd*, but carrying Y10F mutation | This study |
| pQE30-*ugdY71F* | As pQE30-*ugd*, but carrying Y71F mutation | This study |
| pQE30-*ugdY150F* | As pQE30-*ugd*, but carrying Y150F mutation | This study |
| pQE30-*ugdY249F* | As pQE30-*ugd*, but carrying Y249F mutation | This study |
| pQE30-*ugdY335F* | As pQE30-*ugd*, but carrying 335F mutation | This study |
| pQE30-*ugdY380F* | As pQE30-*ugd*, but carrying Y380F mutation | This study |
| pQE30-*wzc_cyto_* | Encoding Wzc cytoplasmic fragment from Ser^447^ to Lys^720^, cloned in *Bam*HI/*Hin*dIII sites, Amp^R^ | Grangeasse *et al*, 2003 |
| pQE30-*etk_cyto_* | Encoding Etk cytoplasmic fragment from Ala^447^ to Glu^726^, cloned in *Bam*HI/*Hin*dIII sites, Amp^R^ | This study |
| pUC18-*rcsA* | pUC18 with a 818-bp *Bam*HI/*Hin*dIII fragment encoding the RcsA transcriptional activator | Vincent *et al*, 2000 |
| pUC18*-ugd*-*rcsA* | pUC18-*rcsA* with a 1167-bp *Sac*I/*Acc65*I fragment encoding entire Ugd _1-388_ protein | This study |
| pUC18-*ugdY71F-rcsA* | pUC18-*rcsA* with a 1167-bp *Sac*I/*Acc65*I fragment encoding entire Ugd _1-388_ protein but carrying Y71F mutation | This study |
| pUC18-*ugd* | pUC18 with a 1167-bp *Acc65*I/*Bam*HI fragment encoding entire Ugd _1-388_ protein | This study |
| pUC18-*ugdY71F* | pUC18 with a 1167-bp *Acc65*I/*Bam*HI fragment encoding entire Ugd _1-388_ protein but carrying Y71F mutation | This study |

References

1. Bullock WO, Fernandez JM, Short JM (1987) A high efficiency plasmid transforming recA *Escherichia coli* strain with beta-galactosidase selection. *Biotechniques* **5:** p376

2. Yanisch-Perron C, Vieira J, Messing J (1985) Improved M13 phage cloning vectors and host strains: nucleotide sequences of the M13mp18 and pUC19 vectors. *Gene* **33:** 103-19

3. Grangeasse C, Obadia B, Mijakovic I, Deutscher J. Cozzone AJ, Doublet P (2003) Autophosphorylation of the Escherichia coli protein kinase Wzc regulates tyrosine phosphorylation of Ugd, a UDP-glucose dehydrogenase. *J Biol Chem* **278 :** 39323-29

4. Vincent C, Duclos B, Grangeasse C, Vaganay E, Riberty M, Cozzone AJ, Doublet P (2000) Relationship between exopolysaccharide production and protein-tyrosine phosphorylation in gram-negative bacteria. *J Mol Biol* **304:** 311-21
