# Supplementary material for: Tyrosine Phosphorylation of the UDP-Glucose Dehydrogenase of Escherichia coli Is at the Crossroads of Colanic Acid Synthesis and Polymyxin Resistance
Source: PLoS One. 2008 Aug 25;3(8):e3053. doi: 10.1371/journal.pone.0003053 (PMC2516531; doi:10.1371/journal.pone.0003053)
Supplement: Table S2 — Primers used in this study (0.04 MB DOC) [file pone.0003053.s003.doc]

**Table S2 . Primers used in this study**

| **Primer**^d^ | **5' to 3' Sequence ^a,b,c,e^** |
| --- | --- |
| pQE30-5’-*ugd-Bam*HI (+) | TAT*GGATCC*AAAATCACCATTTCCGGTACTGGC |
| pQE30-3’-*ugd*-*Hin*dIII  (-) | TAT*AAGCTT*TTAGTCGCTGCCCAAAGAGTCG |
| pQE30-5’-*etk-Bam*HI (+) | TAT*GGATCC*GCGATGTTGCGTCGTGGTG |
| pQE30-3’-*etk*-*Hin*dIII  (-) | TAT*AAGCTT*TTACTCTTTCTCGGAGTAACTATAAC |
| *ugd*-Y10F (+) | TAT*GGATCC*AAAATCACCATTTCCGGTACTGGCT**T**TGTAGGCTTGTC |
| *ugd*-Y71F (-) | CAGCATCCCGG**A**AGGCTTCATTTTTATC |
| *ugd*-Y150F (-) | GGATGGAGATTATCG**A**AAAGGGCTTTACC |
| *ugd*-Y249F (+) | CCGTCGTTTGGTT**T**TGGTGGTTATTGTC |
| *ugd*-Y335F (+) | GAAGTGATCATCT**T**CGAGCCAGTGATG |
| *ugd*-Y380F (-) | TAT*AAGCTT*TTAGTCGCTGCCAAAGAGATCGCGGGTG**A**ATACCTTATCTGCC |
| pUC-5’-*ugd-Acc*65I (+) | CGG*GGTACC*AGGAGGTATAAGA**ATG**AAAATCACCATTTCCGGTACTG |
| pUC-5’-*ugd-Sac*I (+) | TAT*GAGCTC*AGGAGGTATAAGA**ATG**AAAATCACCATTTCCGGTACTG |
| pUC-3’-*ugd*-*Bam*HI (-) | TAT*GGATCC***TTA**GTCGCTGCCCAAAGAGTCG |
| pUC-3’-*ugd-Acc*65I (-) | CGG*GGTACC***TTA**GTCGCTGCCAAAGAGATCG |
|  |  |
| 5’-KO-*ugd* (+) | TGTAAGTAACAAAAGACAATCAGGGCGTAAATAGCCCTGATAACAAGATG***GTGTAGGCTGGAGCTGCTTC*** |
| 3’-KO-*ugd* (-) | GATGCTAAAAACATCATGATTCACAGTTAAGTTAATTCTGAGAGCATGAA***CATATGAATATCCTCCTTAG*** |
| RT-*etk* (-) | CTCTTTCTCGGAGTAACTATAACCG |
| RT-PCR-3’-*etk* (-) | GTTCGGTACGCTGGCTCAGGAC |
| RT-PCR-5’-*etk (+)* | CAGCGGCCTGAGCGATATGATCC |

^a^ Start codons, stop codons and mutated bases are bolded.

^b^ Restriction sites are italicised.

^c^ RBS sequences are underlined.

^d^ Forward and reverse primers are represented by plus (+) or minus (-),respectively.

^e^ Sequences homologous to the Kan^R^ cassette are bolded and italicised.
